# The mitochondrial genome of *Chthamalus malayensis* (Sessilia: Chthamalidae) and its molecular phylogeny within Cirripedia

**DOI:** 10.1080/23802359.2021.1878956

**Published:** 2021-02-17

**Authors:** Sheng Mao, Tian Ge, Yuefeng Cai, Nanjing Ji, Xue Kong, Xin Shen

**Affiliations:** a Jiangsu Institute of Marine Resources/Jiangsu Key Laboratory of Marine Biotechnology, Jiangsu Ocean University, Lianyungang, PR China; b Co-Innovation Center of Jiangsu Marine Bio-industry Technology, Jiangsu Ocean University, Lianyungang, PR China

**Keywords:** Cirripedia, *Chthamalus malayensis*, mitochondrial genome, phylogeny, gene arrangement

## Abstract

Cirripedia is a lower crustacean that has an invaluable place in several aspects of intertidal ecology and anti-fouling research. In this study, we present the first mitochondrial genome of *Chthamalus malayensis*. The complete mitochondrial genome of *C. malayensis* is a circular molecule of 15,230 bp. In comparison to the pancrustacean ground pattern, the mitochondrial genome of *C. malayensis* has a deletion of the *trnC* gene. Phylogenetic analysis based on mitochondrial protein-coding genes showed that *C. malayensis* clusters with *C. antennatus* (BP = 98) and is grouped with *C. challengeri*, *Octomeris* sp. BKKC-2014, and *Notochthamalus scabrosus.* Further studies are needed to reveal the specific phylogenic relationships within Cirripedia.

Cirripedia has an invaluable place in several aspects of intertidal ecology and anti-fouling research. In this study, samples of *Chthamalus malayensis* were collected from the Hainan Islands (N: 20.06, E: 110.35), China. The samples were conserved in the Marine Museum of Jiangsu Ocean University. Total DNA was extracted from the muscle tissues of the samples using the TIANamp DNA Kit (TIANGEN, Beijing, China) according to the manufacturer’s instructions. DNA samples were stored at the Marine Museum of Jiangsu Ocean University (Accession number: Chma-001) and the mitochondrial genome of *C. malayensis* sequenced and annotated according to our previous study (Chen et al. 2019).

The mitochondrial genome is extranuclear genetic material that is maternal inheritance. Mitochondrial DNA can be easily sequenced and is an important molecular marker relating to metazoan phylogeny (Boore and Brown 1998). In this study, the mitochondrial genome of *C. malayensis* is presented. It is a circular DNA molecule containing 15,230 bp (GenBank accession number: MW076458), which encodes 13 protein-coding genes (PCGs), 21 tRNA genes, and 2 rRNA genes (Supplementary Table S1). In particular, a deletion of *trnC* exists compared with the pancrustacean ground pattern. Two PCGs (*cob* and *nd6*) and four tRNAs (*trnF*, *trnS_2_*, *trnT*, and *trnQ*) were found to be located in the light strand and the remaining genes were found to be located in the heavy strand. Seven longer non-coding regions with intergenic sequences more than 30 bp were found. All non-coding regions are 789 bp in length and the longest non-coding region (334 bp) is located between the *12S rRNA* region and the *trnK* gene.

To clarify the phylogenetic relationships within Cirripedia, a phylogenetic tree was constructed based on the amino acid sequences of 13 PCGs from the complete mitochondrial genomes of 30 Cirripedia species (28 Sessilia and 2 Pedunculata) using the PhyloSuite software (Shen et al. 2017; Zhang et al. 2020). As shown in Figure 1, within Chthamalidae, *C. malayensis* clusters with *C. antennatus* into a branch (BP = 98), and then grouped with *C. challengeri* with high support (BP = 100). Furthermore, above-mentioned three species are grouped with *Octomeris* sp. BKKC-2014 and *Notochthamalus scabrosus*, successively. The phylogenetic tree also showed that the Balanidae and Archaeobalanidae cluster together.

**Figure 1.**
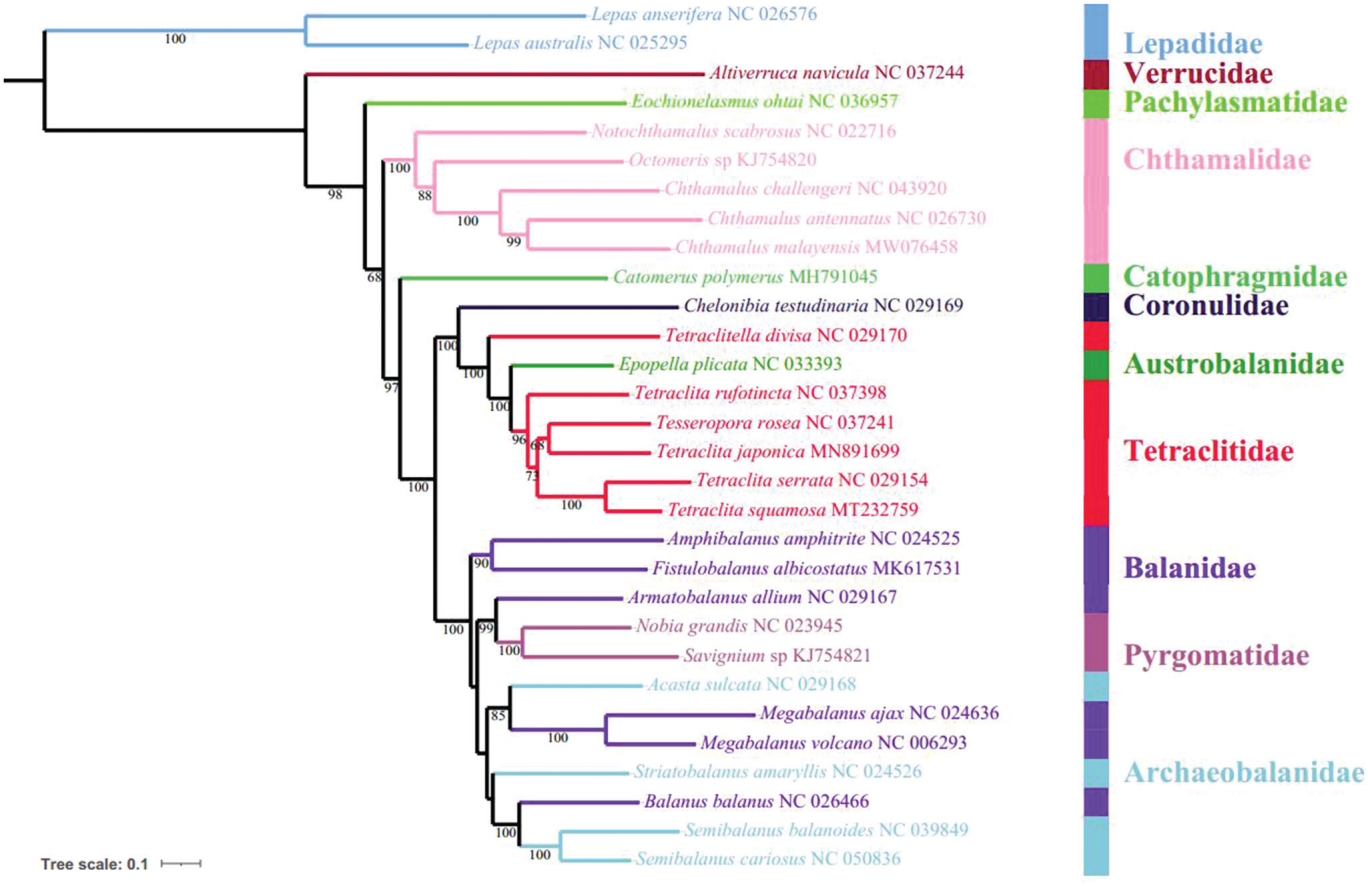
The phylogenetic tree based on 13 PCGs nucleotide acid sequences of *C. malayensis* and other 29 Cirripedia species.

Gene arrangements can help to better understand the phylogenetic relationships among Cirripedia species (Tsang et al. 2017). In comparison to the pancrustacean ground pattern, the mitochondrial genome of *C. malayensis* exhibits massive gene rearrangements (Supplementary Figure S1). In terms of Chthamalidae, *C. antennatus*, *C. challengeri*, and *C. malayensis* share the same gene order, however, *C. malayensis* has a deletion of the *trnC* gene. Moreover, the gene order of *N. scabrosus* was conserved as evidenced by its basal location in Chthamalidae. In this study, we present the first mitochondrial genome of *C. malayensis.* These data can help to better understand the phylogenetic history within Cirripedia, however, more data and further research are needed to reveal the phylogeny within Cirripedia.

## Supplementary Material

Supplemental Material

## Data Availability

The genome sequence data supporting the findings of this study are openly available in GenBank of NCBI at (https://www.ncbi.nlm.nih.gov/) under the accession no. MW076458. The associated BioProject, SRA, and Bio-Sample numbers are PRJNA685939, SRR13266916, and SAMN17101451, respectively.
