# Supplementary material for: The mitochondrial genome of Chthamalus malayensis (Sessilia: Chthamalidae) and its molecular phylogeny within Cirripedia
Source: Mitochondrial DNA B Resour. 2021 Feb 17;6(2):643–4. doi: 10.1080/23802359.2021.1878956 (PMC7894419; doi:10.1080/23802359.2021.1878956)
Supplement: Supplemental Material [file TMDN_A_1878956_SM1290.docx]

Table S1. Mitochondrial genome annotation of *Chthamalus* *malayensis*.

| Gene | Strand | Position | | Nucleotides | Codons | | Anti-codon | Intergenic sequence * |
| --- | --- | --- | --- | --- | --- | --- | --- | --- |
|  |  | Start | Stop |  | Start | Stop |  |  |
| *cox1* | H | 1 | 1536 | 1536 | CGA | TAA |  | 2 |
| *trnL_2_* | H | 1539 | 1606 | 68 |  |  | TAA | 2 |
| *cox2* | H | 1609 | 2292 | 684 | ATG | TAA |  | 0 |
| *trnD* | H | 2293 | 2355 | 63 |  |  | GTC | 0 |
| *atp8* | H | 2356 | 2514 | 159 | ATT | TAA |  | -7 |
| *atp6* | H | 2508 | 3173 | 666 | ATG | TAA |  | 4 |
| *cox3* | H | 3178 | 3966 | 789 | ATG | TAA |  | 60 |
| *trnG* | H | 4027 | 4089 | 63 |  |  | TCC | 0 |
| *nd3* | H | 4090 | 4441 | 352 | ATT | T- |  | 0 |
| *trnR* | H | 4442 | 4504 | 62 |  |  | TCG | 0 |
| *trnN* | H | 4505 | 4567 | 64 |  |  | GTT | 0 |
| *trnA* | H | 4568 | 4630 | 66 |  |  | TGC | 0 |
| *trnE* | H | 4631 | 4695 | 65 |  |  | TTC | 0 |
| *trnS_1_* | H | 4696 | 4753 | 58 |  |  | GCT | 15 |
| *trnF* | L | 4769 | 4832 | 64 |  |  | GAA | 34 |
| *trnK* | H | 4867 | 4931 | 65 |  |  | TTT | 0 |
| control region |  | 4932 | 5265 | 334 |  |  |  | 0 |
| *srRNA* | H | 5266 | 6019 | 754 |  |  |  | 2 |
| *trnV* | H | 6022 | 6087 | 66 |  |  | TAC | 0 |
| *lrRNA* | H | 6088 | 7394 | 1307 |  |  |  | 0 |
| *trnL_1_* | H | 7395 | 7462 | 68 |  |  | TAG | 42 |
| *nd1* | H | 7505 | 8434 | 930 | ATT | TAG |  | 14 |
| *trnY* | H | 8449 | 8513 | 65 |  |  | GTA | 16 |
| *trnS_2_* | L | 8530 | 8599 | 70 |  |  | TGA | -2 |
| *cob* | L | 8598 | 9737 | 1140 | ATG | TAA |  | -1 |
| *nd6* | L | 9737 | 10210 | 474 | ATT | TAA |  | 15 |
| *trnT* | L | 10226 | 10290 | 65 |  |  | TGT | 12 |
| *trnP* | H | 10303 | 10365 | 63 |  |  | TGG | 9 |
| *nd4L* | H | 10375 | 10659 | 285 | ATT | TAA |  | -7 |
| *nd4* | H | 10653 | 11982 | 1330 | ATG | T- |  | 0 |
| *trnH* | H | 11983 | 12047 | 65 |  |  | GTG | 15 |
| *nd5* | H | 12063 | 13751 | 1689 | ATT | TAG |  | 81 |
| *trnI* | H | 13833 | 13899 | 67 |  |  | GAT | 75 |
| *trnQ* | L | 13975 | 14042 | 68 |  |  | TTG | 48 |
| *trnM* | H | 14091 | 14157 | 67 |  |  | CAT | 0 |
| *nd2* | H | 14158 | 15156 | 999 | ATG | TAA |  | -1 |
| *trnW* | H | 15156 | 15221 | 66 |  |  | TCA | 9 |

Note: * Negative numbers indicate overlapping nucleotides between adjacent genes.


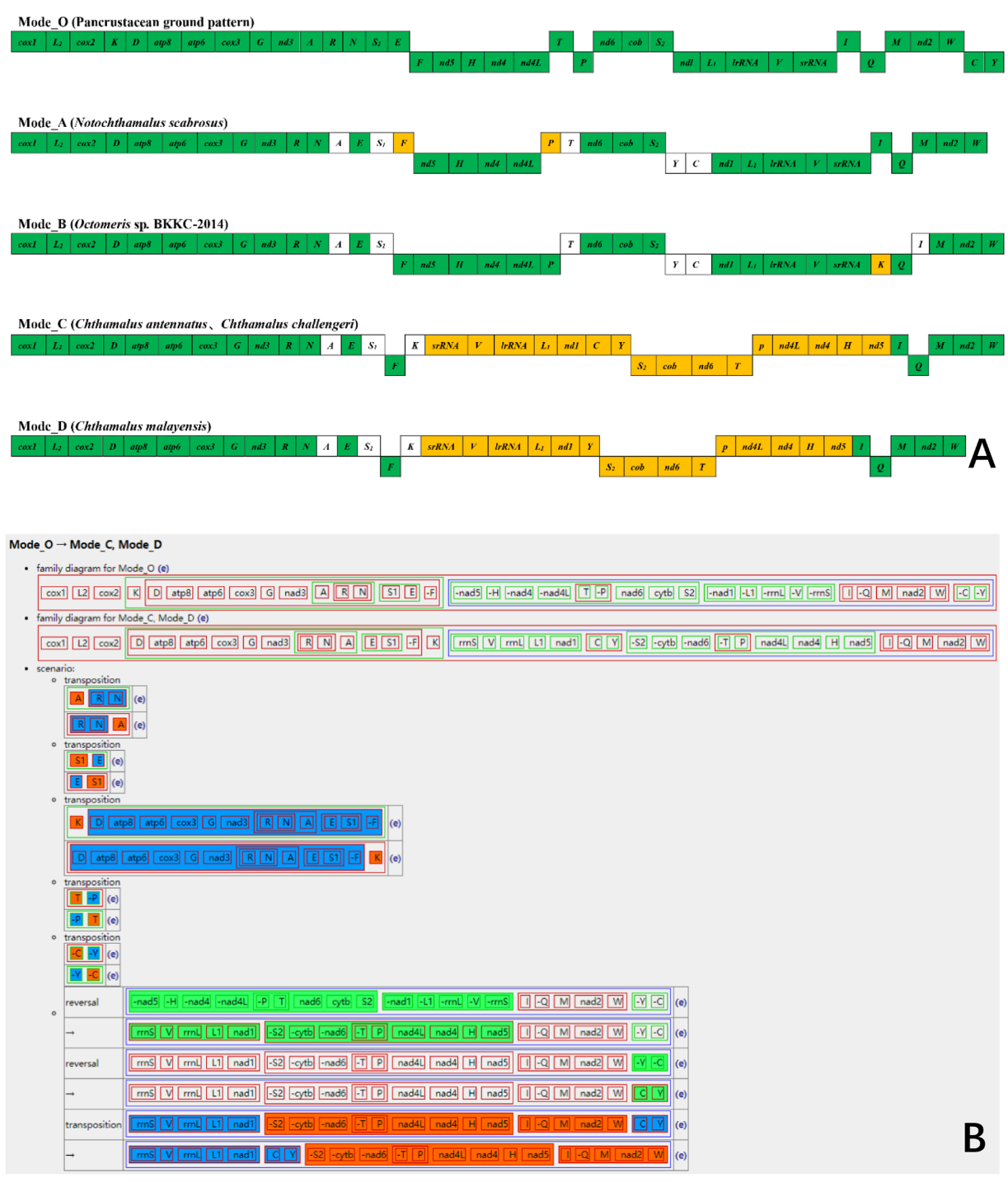


Figure S1. Gene rangements of Pancrustacean ground pattern and five mitochondrial genomes from Chthamalidae which have been classified. (A) Gene rangements of five mitochondrial genomes; (B) Gene arrangements scenario.
